# Radiation enteropathy‐related depression: A neglectable course of disease by gut bacterial dysbiosis

**DOI:** 10.1002/cam4.6865

**Published:** 2024-03-08

**Authors:** Xinliang Liu, Ying Li, Meichen Gu, Tiankai Xu, Chuanlei Wang, Pengyu Chang

**Affiliations:** ^1^ Department of Radiation Oncology and Therapy The First Hospital of Jilin University Changchun China; ^2^ Department of Hepatobiliary and Pancreatic Surgery, General Surgery Center The First Hospital of Jilin University Changchun China

**Keywords:** depression, dysbiosis, gut bacteria, HPA, radiation enteropathy

## Abstract

Radiation enteropathy (RE) is common in patients treated with radiotherapy for pelvic–abdominal cancers. Accumulating data indicate that gut commensal bacteria determine intestinal radiosensitivity. Radiotherapy can result in gut bacterial dysbiosis. Gut bacterial dysbiosis contributes to the pathogenesis of RE. Mild to moderate depressive symptoms can be observed in patients with RE in clinical settings; however, the rate of these symptoms has not been reported. Studies have demonstrated that gut bacterial dysbiosis induces depression. In the state of comorbidity, RE and depression may be understood as local and abscopal manifestations of gut bacterial disorders. The ability of comorbid depression to worsen inflammatory bowel disease (IBD) has long been demonstrated and is associated with dysfunction of cholinergic neural anti‐inflammatory pathways. There is a lack of direct evidence for RE comorbid with depression. It is widely accepted that RE shares similar pathophysiologic mechanisms with IBD. Therefore, we may be able to draw on the findings of the relationship between IBD and depression. This review will explore the relationship between gut bacteria, RE, and depression in light of the available evidence and indicate a method for investigating the mechanisms of RE combined with depression. We will also describe new developments in the treatment of RE with probiotics, prebiotics, and fecal microbial transplantation.

## 1 INTRODUCTION

According to evidence‐based medicine (EBM) data, 52% of cancer patients require radiotherapy (RT) during their treatment course.^1^ Radiation‐induced tissue injury remains a major challenge when performing RT, especially in tissues that are highly sensitive to ionizing radiation (such as the intestinal epithelium).^2^, ^3^ Statistically, the incidence of radiation enteropathy (RE) differs among various tumor types treated with RT, with 80%–90% observed in carcinoma of the cervix, 60% observed in endometrial cancer, and 50% observed in carcinoma of the vulva.^4^ The clinical manifestations of RE typically include acute diarrhea, blood in the stool, chronic intestinal fibrosis, strictures, perforations, and fistulas, among other effects.^5^

The pathogenesis of RE is complicated and typically includes intestinal stem cell apoptosis, endothelial injury, oxidative stress, and inflammation.^6^ In fact, gut bacterial dysbiosis can greatly contribute to these processes. When RE occurs, bacterial overgrowth becomes a characteristic feature.^7^ McLaughlin and Matsuzawa discovered that germ‐free (GF) mice outlived normal mice after abdominal irradiation in 1964, which elicited the study of gut microbiota in RE.^8^, ^9^, ^10^ As research progressed, the relationship between the gut microbiota and intestinal radiosensitivity became clearer.^10^ RT results in gut bacterial dysbiosis.^7^ Gut bacterial dysbiosis can correspondingly increase intestinal radiosensitivity, thus worsening RE.^11^ First, gut bacterial dysbiosis has pro‐inflammatory properties and can increase the levels of inflammatory factors.^12^ Second, gut bacterial dysbiosis can degrade the mucus layer and disrupt the phospholipid bilayer of enterocytes to cause damage to the intestinal mucosa.^13^ The translocation of gut microbiota caused by intestinal epithelial damage exacerbates the intestinal inflammatory response, which correspondingly disrupts the tight junctions between intestinal epithelial cells.^14^, ^15^ Finally, gut bacterial dysbiosis has the ability to cause oxidative stress.^11^ These three processes affect and promote each other, thus forming a feedback loop. In reality, RE is essentially a systemic disease that is mainly due to abnormal metabolism of substances elicited by gut bacterial dysbiosis. Therefore, the restoration of gut bacterial dysbiosis to interrupt this feedback pathway is one of the therapeutic modalities for RE.

Depression is a group of affective psychiatric disorders with a main clinical feature characterized by a marked and persistent depressed mood that is inconsistent with the situation. The pathogenesis of depression is complex, including the monoaminergic transmitter deficiency hypothesis, the neuroinflammatory hypothesis, and HPA axis abnormalities. Recently, several studies have found that gut bacteria play an integral role in the development of depression. Gut bacterial dysbiosis, which is represented by a decline in *Firmicutes* (such as *Lactobacillus*) and an increase in *Bacteroidetes* and *Proteobacteria*, occurs in depressed patients.^16^ The transplantation of feces from depressed mice to healthy mice has been shown to cause depressive behavior in the latter group of mice.^16^ The metabolites of the intestinal flora and the composition of bacteria are key points of this idea.^16^

Depression is often comorbid with a variety of disorders, such as constipation, irritable bowel syndrome, and inflammatory bowel disease (IBD).^17^ Rome IV considered functional gastrointestinal disease to be a disorder of abnormal gut–brain interaction.^18^ It has been reported that 25.2% of patients with IBD present with depressive symptoms.^19^ It can be observed in clinical settings that mild to moderate depressive symptoms are present in RE, and its incidence has not yet been reported. Passive mood increases the risk of recurrence and progression of IBD.^20^ The mechanisms underlying this comorbid relationship are related to abnormalities in the gut–brain axis, including inflammation and autonomic disorders.^21^ In a mouse model of depression, impaired vagus nerve activity increased vulnerability to IBD.^22^ In fact, cholinergic nerves have an anti‐inflammatory role in regulating innate immune cells, which is also known as the “cholinergic anti‐inflammatory pathway”.^23^ After the central nervous system receives the inflammatory signal, it transmits the message to the vagus nerve, thus causing the release of Ach from the vagus nerve endings, which activates the alpha 7 subunit of the nicotinic acetylcholine receptor (α7nAchR) on the membrane of the immune cells and inhibits the synthesis and release of inflammatory factors (such as IL‐6 and TNF‐α) by regulating signaling pathways, such as NF‐kB and JAK/STAT3.^24^ In addition, as early as 1988, a study reported that intravenous injections of choline‐chlorine (a precursor of acetylcholine) prior to radiotherapy improved survival in mice.^25^ Another recent study also confirmed the ability of choline to alleviate doxorubicin‐induced cardiotoxicity by correcting oxidative stress and inflammation.^26^

In recent years, with the development of high‐throughput sequencing technology, it has been found that gut bacteria are a key point in regulating the gut–brain axis; thus, the concept of the “microbiota‐gut‐brain axis” has been proposed.^27^ Gut bacteria interact with the brain through neural, humoral, and immune pathways. The “microbiota‐gut‐brain axis” information exchange is bidirectional. Indeed, in a comorbid state, RE and depression may be understood as being local and distant manifestations of gut bacterial dysbiosis. RE and depression share some similar pathogenetic processes, such as the action of LPS on TLR4, which activates both locally infiltrating macrophages and microglia in the central nervous system, thus exerting pro‐inflammatory effects.^28^ In addition, RE interacts with depression. However, direct evidence is lacking, but we may be able to draw on studies of the relationship between IBD and depression. This review will explore the relationship between gut bacteria, RE, and depression in the context of the available evidence and provide directions for studying the mechanisms of RE combined with depression. We will also describe new developments in the treatment of RE with probiotics, prebiotics, and fecal microbial transplantation.

## 2 GUT COMMENSAL BACTERIA AND RE PATHOGENESIS

### 2.1 Gut commensal bacteria impact intestinal radiosensitivity

The gut microbiota and its metabolites play a considerable role in modulating the growth and function of gut‐associated lymphoid tissues (GALT) and systemic immune systems.^29^, ^30^, ^31^ Preclinical studies have shown that impaired development of lymphoid tissue and regulatory T cells (Treg) were observed in the gut of GF mice.^32^ Nevertheless, all of these abnormalities are reversed after a few weeks of inoculation of GF mice with commensal bacteria.^33^

Intriguingly, subclinical studies have shown that GF mice are more radiation resistant than common mice,^10^ and the mechanism may involve the prominent disability of the intestinal immune response. McLaughlin and Matsuzawa found that GF mice survived significantly longer than common mice after whole‐body X‐ray irradiation.^9^, ^10^ Although Anderson's subsequent studies demonstrated inconsistent results,^34^ several studies in recent years have confirmed Matsuzawa's viewpoint. For example, one study found that GF mice exhibited less apoptosis of endothelial cells when treated with gamma radiation compared to normal mice.^35^ Mechanistically, gut microbiota can downregulate fasting‐induced adipose factor (Fiaf), which is also known as angiopoietin‐like protein 4 (a secreted protein of intestinal epithelial cell origin), which has the ability to promote angiogenesis and the maintenance of mucosal barrier integrity.^35^, ^36^ Similar to the germ‐free mouse assay, Zhao et al. created a near GF mouse intestinal state via pretreatment with antibiotics prior to radiotherapy and found that antibiotic pretreatment reduced the activation of the LPS/TLR4/MyD88/NF‐κB p65 and TGF‐β1/Smad‐3 signaling pathways to attenuate inflammation and reduce intestinal wall fibrosis, respectively, which ultimately mitigated RE.^37^ In summary, the gut microbiota can affect intestinal radiosensitivity by modulating host immunity.

### 2.2 Ionizing irradiation induces gut bacterial dysbiosis

**FIGURE 1:**
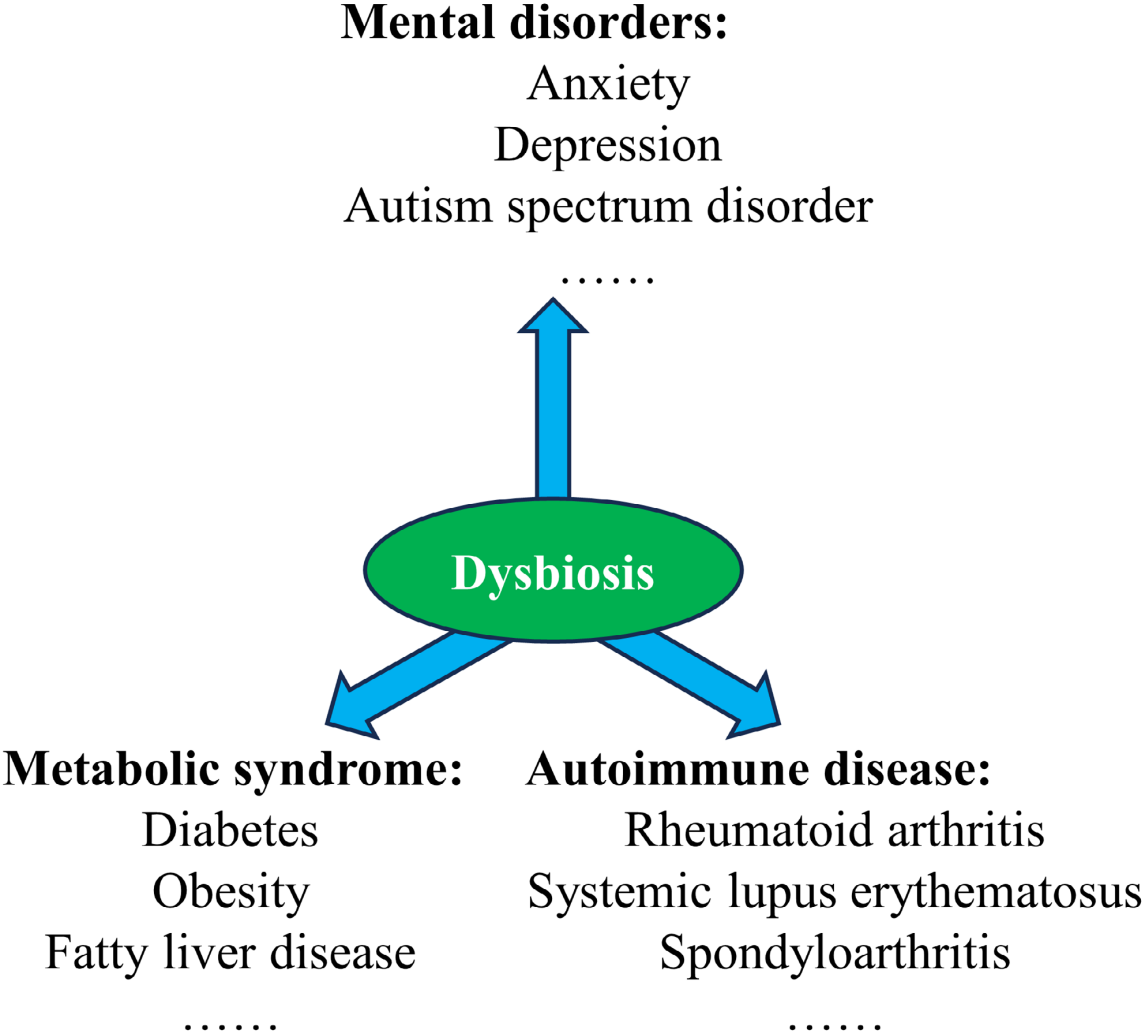
Diseases associated with gut bacterial dysbiosis. Gut bacterial dysbiosis is associated with various diseases, such as mental disorders, metabolic syndromes, and autoimmune diseases.

Perturbations of gut bacteria, which is also known as gut bacterial dysbiosis, have been proven to be associated with various diseases, such as mental disorders, metabolic syndromes such as obesity, and autoimmune diseases (see Figure 1).^38^, ^39^, ^40^ Unfortunately, a multitude of trials have shown that RT induces changes in the composition and function of gut microbiota,^12^, ^41^, ^42^, ^43^ with reduced bacterial diversity^44^ and altered abundance,^13^, ^45^ such as a decrease in the number of probiotics and an increase in the abundance of opportunistic pathogenic bacteria.^46^ Depending on the type of viability and energy metabolism under different oxygen partial pressure conditions, the abundance of specialized anaerobes decreases, whereas parthenogenetic anaerobes, such as *Enterobacteriaceae*, are upregulated.^47^ An early observational study, which included a total of 39 patients undergoing radical pelvic radiotherapy, showed that nearly 25% of patients developed bacterial overgrowth in the small intestine.^7^ Another study showed that pelvic RT resulted in a 10% reduction in *Firmicutes* phyla but a 3% increase in *Fusobacterium* phyla.^45^ Notably, there are no agreed diagnostic criteria for postradiotherapy dysbiosis. Several studies have shown that the degree of gut bacterial dysbiosis is related to the duration and site of irradiation. However, there is a question as to if the degree of dysbiosis is correlated with the irradiation dose. This question merits further investigation.

The intestinal mucosa can secrete antimicrobial substances (also known as defensins) to prevent the overgrowth of pathogenic bacteria.^48^ However, RT causes damage to the intestinal epithelium, which indirectly causes dysbiosis.^6^ Second, different flora have different radiosensitivities to ionizing irradiation,^49^ which may be due to disparities in the content of oxygen radical‐scavenging enzymes in different bacteria.^50^ In addition, several studies have shown that the shaping of gut microbiota by RT is affected by differences in sex and time of treatment.^51^, ^52^

### 2.3 Intestinal radiosensitivity is increased by gut bacterial dysbiosis

**FIGURE 2:**
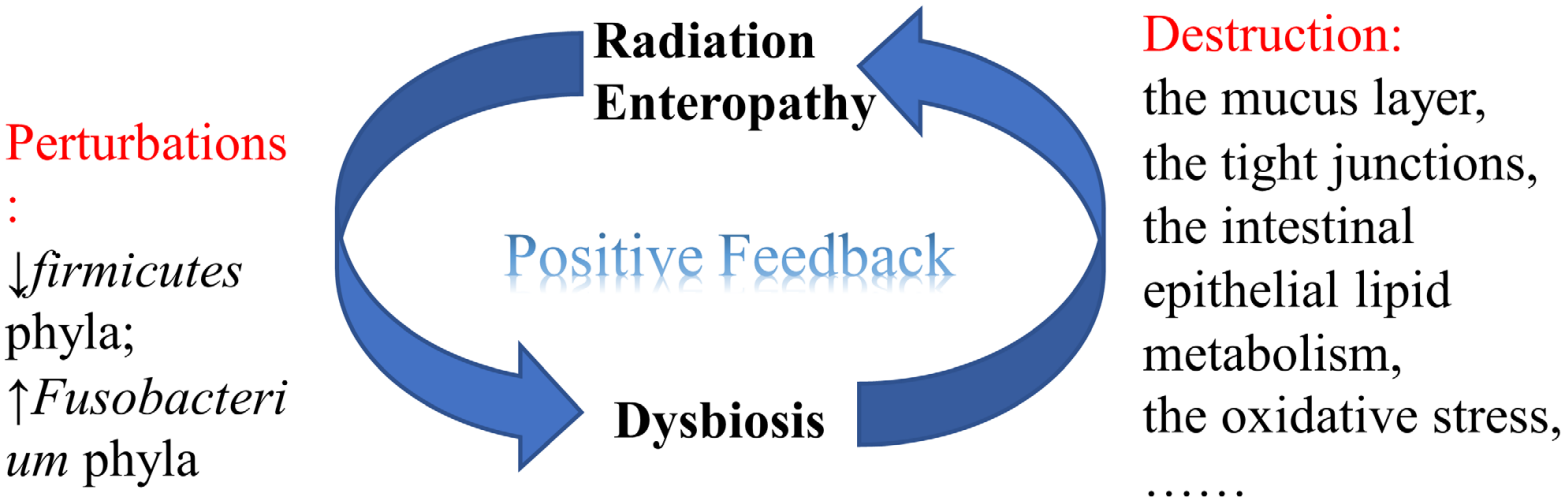
Positive feedback between gut bacterial dysbiosis and radiation enteropathy (RE). Ionizing irradiation induces gut bacterial dysbiosis. Irradiation‐induced dysbiosis is pathogenic, which increases intestinal radiosensitivity and can aggravate RE, constituting vicious positive feedback.

Under normal physiologic conditions, gut commensal bacteria are able to form a “biological barrier” to prevent pathogenic bacteria from colonizing the intestinal epithelium, promote the expression of tight junctions in the intestinal epithelium and the formation of a mucus layer to maintain the integrity of the intestinal epithelial barrier, and increase the expression of anti‐inflammatory genes (such as IL‐10).^53^ However, radiation‐induced gut bacterial dysbiosis is potentially pathogenic, which may increase intestinal susceptibility to irradiation injury^44^ and promote the progression of RE (see Figure 2).

Regarding the mechanism by which radiation‐induced gut bacterial dysbiosis enhances intestinal radiosensitivity, gut bacterial dysbiosis has potential pro‐inflammatory properties. Radiotherapy‐induced intestinal inflammation can alter the composition of the intestinal flora, thus promoting pro‐inflammatory bacteria and inhibiting the growth of anti‐inflammatory bacteria.^54^ For example, *Faecalibacterium prausnitzii* was significantly reduced in a colitis model.^55^ Gerassy‐Vainberg et al.^12^ used an in vitro coculture model of epithelial cells and gut microbiota, in which gut microbiota from irradiated or unirradiated mice were cocultured with epithelial cells in a microaerobic environment. The results showed that the irradiated dysregulated flora significantly upregulated TNF‐α (*p* < 0.05) and IL‐1β (*p* < 0.01) levels in epithelial cells. This finding also demonstrates that FMT can transmit susceptibility to RE and provides evidence that radiation‐induced gut bacterial dysbiosis aggravates RE.^12^ Similarly, in an in vitro monolayer epithelial cell coculture model, Wang et al. found that radiation‐induced dysbiosis can stimulate the release of inflammatory factors such as TNF‐α and IL‐1β.^56^ In addition, the overgrowth of the flora causes reactive aggregation of TH17 cells with pro‐inflammatory properties in the intestine.^57^ Given that the RE phenotype can be transferred through FMT, these experimental results show that gut bacterial dysbiosis may not merely be a result of RE. Instead, these data once again suggest that the gut microbiota may be crucial in the pathogenesis of RE.^58^ Inflammatory signaling pathways between gut bacteria and RE are associated with Toll‐like receptors (TLRs) (see Table 1). Pathogen‐associated molecular patterns (PAMPs) from gut bacteria can be recognized by TLRs, which are one of the most important pattern recognition receptors, on the surface of the body's natural immune cells (such as dendritic cells and macrophages).

**TABLE 1:** Summary of TLRs related to RE.

Receptor	Ligand	Effectivity
TLR2^59^	*Lactobacillus rhamnosus* GG	Improvement of crypt survival rate
TLR4^60^	LPS, peptidoglycan	Upregulation of inflammatory factor expression
TLR5^61^	*Salmonella* flagellin	Radioprotective activity
TLR9^62^	Cytosine‐phosphate‐guanine (CpG) dinucleotides	Radioprotective activity

*Note*: TLR3, TLR7, and TLR8 recognize viral RNAs and are thus not listed.

Second, gut bacterial dysbiosis impairs the intestinal epithelial barrier (IEB). The intestinal mucus layer, the main functional component of which is mucin‐2 (MUC2), is not only involved in constituting the first line of immune defense in the gastrointestinal tract but is also a source of energy for certain commensal bacteria.^63^, ^64^ Studies have shown that gut bacterial dysbiosis interferes with mucus layer formation to increase the contact of gut microbiota with immune cells in the intestinal wall.^13^ In fact, the degradation of mucin significantly increases the risk of developing Crohn's disease.^65^ In addition, radiation‐induced gut bacterial dysbiosis and the disturbance of intestinal epithelial lipid metabolism are related.^66^ The intestinal epithelial cell membrane, which consists of a lipid bilayer, has an indispensable role in maintaining the integrity of the intestinal epithelial barrier.^67^ A recent multiomics study showed that glycerophospholipid metabolism is most correlated with RE and that there is a significant functional correlation between gut microbiota dysbiosis and changes in intestinal epithelial lipid metabolites, such as *Bacteroides*‐diglyceride (DG) (*r* = +0.99) and *Escherichia‐Shigella*‐triglyceride (TG) (*r* = +0.99).^66^ However, there are limitations to this multiomics correlation analysis, which refers to the fact that only a correlation between dysbiosis and lipid metabolites can be demonstrated (not a causal relationship).^66^ Notably, irradiation‐induced dysregulated flora‐induced IL‐1β can disrupt the tight junctions of the intestinal epithelium and aggravate mucosal damage in mice.^14^, ^15^ In addition, a study by Tian et al. found that the waterfall release of TNF‐α is capable of leading to intense oxidative stress and activation of the caspase‐3 pathway, which subsequently induces apoptosis of gut mucosal cells.^68^

Finally, gut bacterial dysbiosis can contribute to oxidative stress.^11^ Oxidative stress is the production of more pro‐oxidative substances, such as reactive oxygen species (ROS), compared to their antioxidative counterparts.^69^ Oxidative stress can be directly induced by the gut microbiota, such as hydroxyl radicals produced by *Enterococcus faecalis* and the release of ROS from intestinal epithelial cells (IECs) promoted by *Peptostreptococcus anaerobius* and *Escherichia coli*.^69^ Conversely, oxidative stress can destabilize homeostasis of the intestinal microbial environment. Notably, inflammatory factors secondary to gut bacterial dysbiosis, such as IL‐1β, IL‐6, and TNF‐α, can promote the aggregation of neutrophils to release ROS (reviewed by Zhang^70^).

Gut bacterial dysbiosis can affect intestinal radiosensitivity through the abovementioned pathways, and there may be interactions between the different pathways. Taken together, irradiation‐induced dysbiosis is pathogenic, which correspondingly increases intestinal radiosensitivity.^54^

## 3 ASSOCIATION BETWEEN DEPRESSION AND GUT MICROBIOTA

Neufeld found that GF mice had less depressive behavior than specific pathogen‐free (SPF) mice,^71^ thus suggesting a nonnegligible role of the gut microbiota in depression. As with RE, there is gut bacterial dysbiosis in depression.^16^, ^72^ For example, *Bifidobacteria*, *Dialister*, and *Coprococcus* spp. were significantly reduced, whereas *segmented filamentous bacteria* (SFB) were increased.^73^ Table 2 below depicts a summary of the changes in gut microbiota in depressed patients.

**TABLE 2:** Alterations in the gut microbiota of depression.

Refs.	Findings		
	Enriched in cases	Reduced in cases	Enriched in controls
^74^	Phyla: *Bacteroidetes*, *Proteobacteria*, and *Actinobacteria*; Genera: *Enterobacteriaceae* and *Alistipes*	Phyla: *Firmicutes*; Genera: *Faecalibacterium*	NA
^73^	NA	Genera: *Coprococcus* and *Dialister*	NA
^75^	Phylum: *Bacteroidetes*, *proteobacteria*, and *Fusobacteria*	NA	Phylum: *Firmicutes* and *Actinobacteria*
^76^	*Eggerthella*, *Atopobium*, and *Bifidobacterium*	*Faecalibacterium*	NA
^77^	NA	*Bifidobacterium*, *Lactobacillus*	NA
^78^	*Janthinobacterium* (blood)	*Neisseria* (blood)	NA

*Note*: Longitudinal heading Ref. [79].

One of the main traits of depression is abnormal immunologic activity. The immune system regulates neurologic growth and development and maintains normal physiologic functions (e.g., the ability of mast cells to synthesize nerve growth factors and the impaired memory and learning abilities of mice lacking T cells).^80^ However, autoimmune diseases exhibit a higher risk of depression; for example, a previous study reported of a 43% increase of depression in Graves' disease.^81^ A meta‐analysis of longitudinal studies also found that elevated inflammatory markers (including C‐reactive protein [CRP] and IL‐6) were significantly associated with an increased risk of subsequent depressive symptoms, thus lending credence to the inflammation‐to‐depression causal link.^82^

The gut microbiota can regulate inflammation to influence depression. The study by Grigoleit demonstrated that LPS increases depressed mood due to excessive release of pro‐inflammatory cytokines.^83^ Moreover, Beurel et al. found that SFB induces depression‐like behavior.^72^ Specifically, SFB is able to secrete autoinducer‐2 (AI‐2, which is a quorum‐sensing molecule) to promote T helper 17 (Th17) cell proliferation and aggregation in the hippocampus. Additionally, a previous study confirmed the antidepressant effect of *Bifidobacteria infantis*.^84^ *B. infantis* reduced the release of pro‐inflammatory cytokines such as IL‐6, IFN‐γ, and TNF‐α and decreased the IFN‐γ:IL‐10 ratio in Sprague–Dawley rats receiving immune stimulation.^84^ Additionally, the *B. infantis* group had significantly higher concentrations of tryptophan and kynurenic acid (Kyna) in the plasma than in the control group.^84^ Wong et al. also showed that it is feasible for the gut microbiota to influence depression by modulating inflammasome signaling.^85^ Recently, Luo et al. found that the depletion of gut bacteria after irradiation reduces the synthesis of brain pro‐inflammatory factors.^86^ In addition, TLRs are distributed in neurons and glial cells, and their mediated signaling pathways are involved in the development of the nervous system and the process of disease development (reviewed by Okun).^87^ For example, the expression of TLR4 mRNA and the synthesis level of downstream proteins are significantly increased in patients with depression.^88^

Several studies have observed differences in the gut microbiota metabolites in depressed patients compared to healthy individuals. For example, the metabolomics of depressed mice was significantly disordered in the blood and feces, with enhanced carbohydrate metabolism and decreased glutamate‐ and phenylalanine‐related metabolites being observed.^16^ Valles‐Colomer found a positive correlation between 3,4‐dihydroxyphenylacetic acid levels and mental health, as well as increased levels of both GABA and glutamate, in patients with depression.^73^ In fact, the gut microbiota can synthesize neuroactive metabolites associated with depression and influence its development. The serotonin concentration in the serum of conventional mice was 2.8 times (*p* = 1.27 × 10^−10^) higher than that of GF mice.^89^ Higuchi reported that *Lactobacillus* sp. *Strain E1* was able to decarboxylate glutamate to form gamma‐aminobutyric acid (GABA).^90^ In addition, the gut microbiota can alter the permeability of the blood–brain barrier (BBB).^91^

In addition to the blood pathway, gut bacterial metabolites can act indirectly on the central nervous system via the autonomic nervous system. Butyrate, which is a metabolite of fermentable carbohydrates, is able to activate vagal neurons and their projecting nucleus medial nucleus tractus solitaries.^92^ In fact, the vagus nerve is the primary pathway by which gut bacteria communicate information to the brain. The *Lactobacillus strain* regulates the expression of GABA (Aα2) mRNA in different regions of the brain, thereby improving depression‐related behaviors.^93^ However, this neurochemical and behavioral effect disappeared after dissecting the vagus nerve.^93^ Moreover, Wang et al. found that subdiaphragmatic vagotomy was able to block the depressive phenotype induced by *Lactobacillus intestinalis* and *Lactobacillus reuteri*.^94^

Stressors have been shown to induce depressive‐like behaviors through mechanisms including the aberrant activation of inflammation and HPA hyperactivity.^95^ In fact, gut bacterial dysbiosis is an integral part of the abovementioned process, which is related to the “leaky gut” hypothesis.^96^, ^97^ Restraint stress leads to hyperactivity of the HPA axis, which is manifested by elevated plasma corticosterone concentrations.^98^ The hyperactive HPA causes an increase in intestinal permeability and the consequent translocation of gut bacteria.^99^ Blood‐entered LPS activates microglial TLR 4 in the brain, thus inducing depressive‐like behaviors by eliciting excessive inflammatory responses.^96^ When cognitive–behavioral therapy was administered, patients with major depressive disorder had a significant decrease in TLR4 mRNA and protein, which was accompanied by a significant improvement in clinical symptoms.^100^ There is a bidirectional regulation between gut bacteria and the HPA axis. Following the administration of restraint stress, GF mice exhibited a hyperactive HPA axis characterized by higher plasma ACTH and corticosterone levels compared to SPF mice.^101^ Meanwhile, the colonization of *Bifidobacterium infantis* into GF mice reversed this result.^101^ Second, stressor exposure disrupts the composition of gut bacteria, such as increasing colonization by the pathogens *Citrobacter rodentium* and *Enterobacteriaceae*, as well as decreasing the genus *Bacteroides*, thus resulting in the upregulation of inflammatory factors.^102^, ^103^ The mechanism of this effect is associated with a decrease in alpha‐defensin secretion by PAN cells and an increase in apoptosis of CD45+CD90+ cells, which produce protective IL‐22.^104^, ^105^ Bailey et al. discovered that antibiotic‐treated mice exposed to a social stressor did not exhibit an increase in IL‐6 or MCP‐1.^106^ This implies that the stressor affects the immune system by altering the intestinal flora composition.^106^

In summary, the three pathways described above interact with each other.^27^ Stimulation of the vagus nerve increased the expression of corticotropin‐releasing factor mRNA in the hypothalamus and plasma levels of corticosterone and corticosterone by upregulating IL‐1 beta expression in the brain.^107^

## 4 GUT BACTERIAL DYSBIOSIS: A PATHOPHYSIOLOGIC LINK BETWEEN DEPRESSION AND RE

**FIGURE 3:**
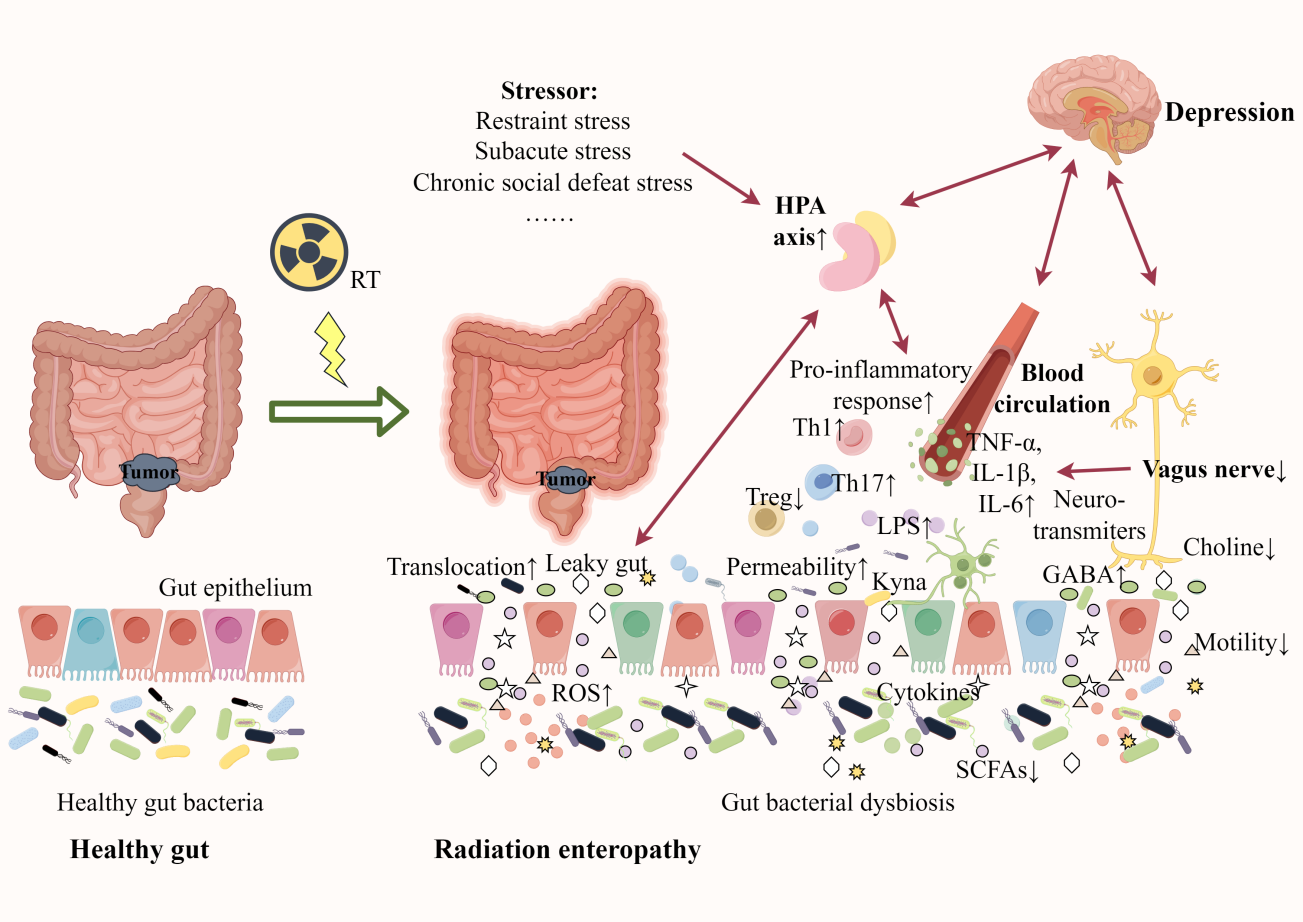
The pathophysiologic link between depression and radiation enteropathy (RE) through gut bacterial dysbiosis. In the state of comorbidity, RE and depression may be understood as being local and distant representations of gut bacterial dysbiosis. The mechanisms underlying this comorbid relationship are related to abnormalities in the gut–brain axis, including inflammation, HPA axis hyperactivity and autonomic disorders.

In the state of comorbidity, RE and depression may be understood as being local and distant representations of gut bacterial dysbiosis (see Figure 3). Gut bacterial dysbiosis can cause changes in intestinal permeability, thus allowing the gut microbiota to enter the bloodstream. The nuclear transcription factors NF‐kB and the NLRP3 inflammasome are activated as a result of the action of PAMPs from translocated gut microbiota, such as LPS and flagellin, on pattern recognition receptors (PRRs) on intrinsic immune cells. The fact that inflammatory factors such as IL‐1, IL‐6, and TNF‐ɑ are increased in both depression and RE is easily understood.^108^ However, there is little direct evidence on whether RE affects depression through inflammation; however, we reasoned this mechanism by examining existing studies of depression in IBD. Bercik et al. have shown that chronic enteritis can induce anxiety‐like behavior, which is a process mediated by pro‐inflammatory cytokines.^109^ One notable aspect of this study is that supplementation with *Bifidobacterium longum* was able to reverse the abnormal behavior. Yang et al. found that intraperitoneal injection of Fast Green FCF inhibited the LPS/TLR4/Myd88/NF‐κB signaling pathway, which attenuated depressive‐like behavior.^110^ Recently, Hu et al. found that oral administration of quercetin to a radiation‐induced brain injury (RIBI) mouse model resulted in improved anxiety, behavioral activity, and memory, as well as decreased TNF‐α and IL‐6 levels, along with reduced intestinal epithelial damage.^111^ These effects disappeared when antibiotics were administered to RIBI mice, thus suggesting that quercetin acts by regulating gut bacteria.^111^

AlSharari et al. found that in DSS‐induced colitis, α7 knockout mice had more severe colitis and higher levels of TNF‐α than wild‐type mice. Choline supplementation (α7nAChR receptor agonist) alleviated DSS‐induced colitis.^112^ Recently, Li et al. elucidated the mechanisms involved from the perspective of gut bacteria, thus confirming that choline mediates intestinal immune tolerance through the activation of α7nAChR.^113^ Specifically, *Enterobacter ludwigii* is able to produce choline, which promotes DC synthesis and the release of retinoic acid (RA) and transforming growth factor beta (TGF‐β). Moreover, RA and TGF‐β are able to induce the differentiation of naïve CD4+ T cells into Treg cells rather than pro‐inflammatory Th1 or Th17 cells.^113^ Choline levels are significantly decreased in intestinal tissue and serum samples from IBD patients.^114^, ^115^ Indeed, cholinergic anti‐inflammatory pathways play an important role in depression‐induced intestinal inflammation.^116^ Ghia et al. demonstrated impaired parasympathetic function and colitis in depressed mice.^22^ Antidepressants were able to prevent or treat depression‐associated intestinal inflammation, but the protective effect was completely lost when the vagus nerve was dissociated.^22^ Another study by these researchers showed that depression can cause chronic to recurrent depression.^21^ Macrophages in blood samples from depressed mice showed abnormal pro‐inflammatory activity, including increased secretion of pro‐inflammatory cytokines.^21^ The mechanism for this effect is that the vagus nerve has an abnormal anti‐inflammatory pathway mediated through α7nAChR.^21^ After a systematic search, no studies have been reported on choline changes in RE.^25^ However, the abovementioned studies may be able to indicate the manner of researching the mechanism of the link between RE and depression.

## 5 MODIFICATION OF GUT MICROBIOTA AND RE MANAGEMENT

In the early 1900s, Russian embryologist Elie Metchnikoff proposed the therapeutic concept that changing the gut microbiota would prolong life.^117^ Mitra et al. found that the severity of radiation‐induced intestinal toxicity was negatively correlated with gut microbiota diversity.^118^ Therefore, researchers have attempted to modulate the composition of the patient's gut microbiota to prevent or alleviate clinical symptoms of RE,^119^ such as RID.^120^

### 5.1 Fecal microbiota transplantation (FMT)

FMT is the transfer of fecal bacteria from a healthy donor to a patient for the treatment of intestinal and extraintestinal diseases via injections of liquid flora or oral administration of flora capsules.^121^ In the late fourth century AD, the use of fecal transplants for the treatment of diseases was documented in the “*Zhou Hou Bei Ji Fang*” written by the Chinese scholar Ge Hong.^122^ In 1988, a clinical trial demonstrated that live *Lactobacillus acidophilus* cultures could be used to prevent RID.^123^ Animal studies have reported of improved intestinal function and epithelial integrity, as well as significant increases in survival and body weight, in mice that received FMT after total body irradiation (TBI).^124^ qPCR assays further confirmed that FMT upregulated small intestinal Vegf mRNA expression levels and promoted angiogenesis without accelerating tumor growth.^124^ Chen et al. found that FMT attenuated radiotherapy‐related toxicity by maintaining PGF2α levels.^125^ On November 30, 2022, the FDA (U.S. Food and Drug Administration) approved the first fecal bacteria product (Rebyota) for the prevention of *Clostridium difficile* infection (CDI) recurrence in people 18 years and older, which was a significant discovery (News from FDA). More indications are likely to be approved in the future.

### 5.2 Probiotics and prebiotics

The effect of probiotics on RE has long been proven. Chitapanarux et al. conducted a prospective, randomized controlled clinical trial (*n* = 63) that investigated the role of *L. acidophilus* and *Bifidobacterium bifidum* in reducing the incidence of acute RID and found a reduction in the incidence and severity of diarrhea, an increase in stool consistency, a significant reduction in antidiarrheal medication use, and no adverse effects in the probiotic group.^126^ Another large double‐blind, placebo‐controlled clinical trial showed that the highly effective probiotic agent VSL#3 (with probiotic lactobacilli as the main ingredient) could reduce the incidence and severity of diarrhea and the use of the antidiarrheal drug loperamide.^127^ A summary of clinical studies on the treatment of RE from a probiotic perspective is shown in Tables 3 and 4.

**TABLE 3:** Summary of published clinical studies on probiotics for radiation enteropathy.

Author, Year	Probiotics/Prebiotics	Type of cancer	Radiation dose	Major findings
Giralt 2008^128^	*Lactobacillus*	Cervical carcinoma, endometrial adenocarcinoma	45–50 Gy	Significantly improved stool consistency, but did not reduce the incidence of diarrhea (Grade 2–5)
Chitapanarux 2010^126^	*Lactobacillus acidophilus* + *Bifidobacterium bifidum*	Cervical cancer	200 cGy	↓ The incidence of diarrhea and the need for anti‐diarrheal medication
Demers 2014^129^	*Bifilact*	Pelvic cancer	40–50.4 Gy	↓ The incidence of diarrhea (Grade 2–4)
Garcia‐Peris 2016^130^	*Inulin, fructo‐oligosaccharide*	Gynecologic cancer	NA	↑ The consistency of stools
Sasidharan 2019^131^	Resistant starch	Cervical cancer	50 Gy	No significant gain
Delia 2007^127^	VSL#3	Sigmoid, rectal, or cervical cancer	60–70 Gy	↓ Incidence and severity of RID, number of bowel movements per day
Linn 2019^132^	*Lactobacillus*	Cervical cancer	50 Gy	↓ Incidence and severity of RID
Urbancsek 2001^120^	*Lactobacillus rhamnosus*	Abdominopelvic tumor	50 Gy	↓ Number of bowel movements

**TABLE 4:** Summary of ongoing clinical studies on probiotics for radiation enteropathy (date from https://clinicaltrials.gov).

NCT number	Study designs	Phases	Start date	Interventions	Outcome measures	Enrollment
NCT03978949	Randomized, double, parallel assignment	III	2019	*Bacillus licheniformis*	Grade 2 or more acute intestinal toxicities	248
NCT05406882	Randomized, open label, crossover assignment	II/III	2022	*Bifidobacterium* and *Lactobacillus*, *Glutamine*	Incidence of radiation proctitis	176
NCT03742596	Non‐randomized, quadruple, parallel assignment	II/III	2018	*Lactobacillus*, *Bifidobacteria*	The level of immunoglobulin (Ig) A	40
NCT03516461	Non‐randomized, open label, parallel assignment	NA	2018	FMT	Change of toxicity grade	30
NCT05032027	Randomized, quadruple, intervention model: Parallel assignment	NA	2021	Probiotics	Grade 3 enteritis	40

As gene editing technology becomes more accurate and affordable (especially with the development of CRISPR technology), engineered probiotics are becoming more feasible and widespread.^133^ Compared with conventional probiotics, engineered probiotics have better specificity and safety.^133^ The anti‐inflammatory cytokine IL‐22 fosters repair and healing of the intestinal mucosa.^134^ IL‐22‐producing recombinant next‐generation probiotics (NGPs), which are also known as live biotherapeutic products (LBPs),^135^ have been shown to increase 30‐day survival in C57BL/6 mice undergoing TBI by up to 85%.^136^

Probiotics are defined as substrates that can be selectively utilized by host microorganisms and provide a benefit to the host.^137^ Some studies have confirmed that prebiotics have the ability to promote the proliferation and replication of probiotics and consequently improve quality of life in abdominopelvic tumor patients.^131^, ^138^ A randomized, double‐blind, placebo‐controlled clinical trial in gynecologic oncology patients treated with postoperative abdominal radiotherapy included 38 patients, with the experimental group given inulin and fructo‐oligosaccharide and the control group receiving maltodextrin. The results of this study showed that prebiotics administered in the experimental group restored the number of corresponding probiotics more quickly, reduced the number of days of watery stools (*p* = 0.07), and improved the quality of life of patients undergoing abdominal radiotherapy.^130^

### 5.3 Antibiotics

The overproliferation and translocation of gut bacteria is one of the characteristics of RE. Therefore, antibiotics are one of the options for the treatment of RE‐related infections.^139^ The *Chinese consensus on the diagnosis and treatment of radiation proctitis* (2018) states that antibiotics can be given to patients with RE who have uncomfortable symptoms, such as abdominal bloating and diarrhea due to bacterial overproliferation; moreover, the course of treatment is usually 7–10 days (Grade of recommendation: 1B).^140^ The efficacy of different antibiotics in the treatment of bacteremia secondary to RE was systematically described earlier by Brook et al.^140^ The palliative effect of metronidazole in chronic RE was confirmed in a study by Cavcić et al.^141^ Recently, Cui et al. found that the survival rate of the antibiotic‐treated group was significantly higher than that of the blank control group in mice after 6.5 Gy irradiation.^142^

When using antibiotics, their safety should not be ignored, especially their effects on antitumor efficacy. Some antibiotics can directly exert anticancer effects through the following mechanisms: antiproliferative, proapoptotic, and anti‐epithelial mesenchymal transition (EMT) mechanisms (reviewed by Gao).^143^ For example, vancomycin (an antibiotic that acts primarily on Gram‐positive bacteria and that has antimicrobial action limited to the gut) enhances RT‐induced antitumor immune responses and tumor growth inhibition. However, Gopalakrishnan et al. found that the administration of antibiotics before and after immunotherapy shortened progression‐free survival and overall survival in patients with stage IV tumors. In addition, vancomycin has been reported to impair intestinal IL‐17A and GM‐CSF‐mediated antifungal immune responses, thereby increasing the risk of fungal infections. Brook et al. found that an increase in lactose‐fermenting bacteria was associated with septicemia mortality, which was associated with the use of broad‐spectrum anti‐anaerobic agents.^144^ This study demonstrates the need for precision antimicrobial therapy.

Finally, antibiotics are effective and relatively specific in killing intestinal flora. Therefore, antibiotics serve as a widely used experimental intervention, both in basic and clinical trials related to RE.

### 5.4 Other

In recent years, a number of new radiation protection agents have been explored in basic research. According to a rodent study, the green tea polyphenol (−)‐epigallocatechin‐3‐gallate (EGCG) was found to protect against RE by increasing the ratio of *Firmicutes/Bacteroidetes* and the abundance of probiotics, thereby reverting gut dysbiosis.^145^ Zhang et al. found that urolithin A (UroA) improved intestinal cell regeneration, which was primarily accomplished through the restoration of the intestinal bacterial profile.^146^ Li et al. found that butyrate attenuated RE via the activation of G protein‐coupled receptors (GPCRs) and the restoration of the intestinal bacterial composition.^147^ Another study by Li et al. found that 30% calorie‐restricted dietary preconditioning ameliorated radiation enterotoxicity, which was associated with a reduced abundance of pro‐inflammatory bacteria and increased numbers of short‐chain fatty acid‐producing bacteria.^148^ Recently, Wang et al. found that orally administered carbon nanoparticles can remove ROS, prevent gut bacterial dysbiosis, and reduce the apoptosis of intestinal epithelial cells to play a role in radiation protection.^149^

## 6 CONCLUSION

Gut commensal bacteria determine intestinal radiosensitivity. Ionizing irradiation induces gut bacterial dysbiosis, which increases intestinal radiosensitivity and induces depression. Patients with RE combined with depression tend to have a poorer prognosis. In the state of comorbidity, RE and depression may be understood as being local and abscopal manifestations of gut bacterial disorders. The microbiota–gut–brain axis is a potential mechanism. Gut bacteria is a potential target for the treatment of RE. Current or pending problems in the field include patients with RE with comorbid depression who are not properly identified and valued. Both preclinical and clinical evidence lacks consistent, high‐quality conclusions about the therapeutic effects of gut microbiota and metabolites. Complete control of confounding factors is not easy because the gut microbiota is susceptible to a variety of factors. In addition, most studies on gut microbiota and disease have been cross‐sectional studies, yet this only yields correlations and lacks longitudinal studies. Moreover, most of the studies have been conducted in animals, and there is still much work to be done to translate the results of animal tests to complex humans. There are several future research directions that can be enacted. First, the characterization of the gut microbiota before radiotherapy can be used to assess and predict gastrointestinal syndromes after radiotherapy, which can correspondingly be stratified by risk factors and screened for high‐risk groups, thus helping clinicians in developing individualized treatment plans. In the intestine, in addition to bacteria, fungi, viruses, archaea, and other microorganisms interact with each other and constitute an intricate grid. Therefore, the study of RE cannot be limited to the bacterial level. At present, the study of intestinal flora is still in the initial stage, and with the introduction of more new and efficient research methods (such as Mendelian randomization analysis and artificial intelligence algorithms), it will greatly facilitate the development of this field.

## AUTHOR CONTRIBUTIONS

**Xinliang Liu:** Writing – original draft (lead). **Ying Li:** Formal analysis (supporting). **Meichen Gu:** Formal analysis (supporting). **Tiankai Xu:** Supervision. **Chuanlei Wang:** Methodology (equal). **Pengyu Chang:** Conceptualization (equal); funding acquisition (equal); writing – review and editing (equal).

## FUNDING INFORMATION

This article was supported by National Natural Science Foundation of China under grant number: 82272738, 81874254; Scientific and Technological Developing Scheme Foundation of Jilin Province under grant number: 20200201400JC; and Special Foundation to health professionals of Jilin Province under grant number: JLSWSRCZX2020‐00931. The Figure 3 in this review are made by Figdraw (www.figdraw.cosssm).

## CONFLICT OF INTEREST STATEMENT

The authors declare that there is no conflict of interest. This article has been read and approved for publication in the journal by all authors.

## Data Availability

Not applicable.
